# Methods for costing malaria service delivery using secondary data

**DOI:** 10.1186/1475-2875-11-S1-P32

**Published:** 2012-10-15

**Authors:** Katva Galactionova, Fabrizio Tediosi, Konstantina Boutsika

**Affiliations:** 1 Swiss Tropical and Public Health Institute, Basel, CH-4002, Switzerland; 2 University of Basel, Basel CH-4003, Switzerland

## 

Malaria is one of the major public health problems for low income countries, a major global health priority, one that carries a dramatic economic impact. Funding and consequently coverage of both preventive interventions and of case management for malaria control have been rising and both international donors and governments of malaria endemic countries need the tools and evidence to assess which are the best and most efficient strategies. To aid with these decisions we developed an open access malaria costing database that effectively summarizes an extensive body of literature on costing and effectiveness of malaria preventive interventions and case management. The database comprises 150 publications spanning from 1985 through 2012 from a total of 42 countries. We collected costs, detailed site and intervention data on insecticide residual spraying campaigns, insecticide treated net distribution and re-treatment campaigns, delivery of intermittent preventive treatment, and treatment of uncomplicated and severe malaria. Unit costs and other intervention data were coded into standardized categories and expressed in common 2008 international dollars allowing us to compare economic outlays across sites and interventions. We discuss findings from these descriptive analyses and illustrate how these data can be used to extrapolate costs to other countries.
